# Antitumor Effects and Delivery Profiles of Menahydroquinone-4 Prodrugs with Ionic or Nonionic Promoiety to Hepatocellular Carcinoma Cells

**DOI:** 10.3390/molecules23071738

**Published:** 2018-07-16

**Authors:** Shuichi Setoguchi, Daisuke Watase, Kazuhisa Matsunaga, Hirofumi Yamakawa, Shotaro Goto, Kazuki Terada, Kenji Ohe, Munechika Enjoji, Yoshiharu Karube, Jiro Takata

**Affiliations:** Faculty of Pharmaceutical Sciences, Fukuoka University, Nanakuma, Jonan-ku, Fukuoka 814-0180, Japan; ssetoguchi@fukuoka-u.ac.jp (S.S.); watase@fukuoka-u.ac.jp (D.W.); hyamakawa@adm.fukuoka-u.ac.jp (H.Y.); sgoto@fukuoka-u.ac.jp (S.G.); kterada@fukuoka-u.ac.jp (K.T.); ohekenji@fukuoka-u.ac.jp (K.O.); enjoji@adm.fukuoka-u.ac.jp (M.E.); karube@fukuoka-u.ac.jp (Y.K.); jtakata@fukuoka-u.ac.jp (J.T.)

**Keywords:** prodrug, menahydroquinone-4, menaquinone-4, drug delivery system, hepatocellular carcinoma

## Abstract

Hepatocellular carcinoma (HCC) shows poor prognosis owing to its very frequent recurrence even after curative treatment. Thus, an effective and safe long-term chemopreventive agent is strongly in demand. Menahydroquinone-4 (MKH) is an active form of menaquinone-4 (MK-4, vitamin K_2_) that is involved in the synthesis of vitamin K-dependent proteins in the liver. We hypothesized that efficient delivery of MKH might be critical to regulate HCC proliferation. The discovery of a suitable prodrug targeting HCC in terms of delivery and activation could reduce the clinical dose of MK-4 and maximize efficacy and safety. We previously showed that MKH dimethylglycinate (MKH-DMG) enables effective delivery of MKH into HCC cells and exhibits strong antitumor effects compared with MK-4. In this study, we prepared anionic MKH hemi-succinate (MKH-SUC) and non-ionic MKH acetate (MKH-ACT), in addition to cationic MKH-DMG, and evaluated MKH delivery profiles and antitumor effects in vitro. MKH-SUC showed the highest uptake and the most efficient release of MKH among the examined compounds and exhibited rapid and strong antitumor effects. These results indicate that MKH-SUC might have a good potential as an MKH delivery system for HCC that overcomes the limitations of MK-4 as a clinical chemopreventive agent.

## 1. Introduction

Hepatocellular carcinoma (HCC) is one of the most common human malignant tumors in the world. Despite so-called curative treatments, such as surgical resection or liver transplantation, the long-term prognosis of HCC is very poor because of a high relapse rate and frequent incidence of intrahepatic metastasis [1,2]. Sorafenib, a vascular endothelial growth factor receptor tyrosine kinase inhibitor, is the first systemic agent to be approved for the treatment of unresectable HCC, and regorafenib and nivolumab have also been approved [3,4]. More recently, in Japan, lenvatinib has been approved for the first time in the world. These systemic agents are excellent developments in the treatment of HCC; however, these agents are only applicable for unresectable HCC, and strong adverse effects, such as skin and liver toxicities, hemorrhage and hypertension, are sometimes observed [1,3]. Therefore, less toxic and effective agents for HCC at an earlier stage are needed.

A number of findings have shown that menaquinone-4 (MK-4, vitamin K_2_) may play a role in suppressing the growth and recurrence of HCC both in vitro and in vivo [5,6,7,8,9,10]. MK-4 has been used for osteoporosis, in particular, for postmenopausal women, and therefore, its long-term safety has been established [11,12,13,14,15]. MK-4 was speculated to be an ideal adjuvant agent if it could reduce the cumulative recurrence of HCC by preventing *de novo* carcinogenesis or suppressing tumor growth in a clinical trial. However, a recent larger scale, double-blind, randomized, placebo-controlled trial in Japan did not find any statistically significant improvement due to MK-4 on the cumulative recurrence of HCC at a clinical dose (45 mg/day) or double dose (90 mg/day) for osteoporosis [16].

Previous studies showed that the levels of vitamin K in HCC tissues are lower than those in the surrounding non-tumorous tissues and, in particular, MK4-10 concentrations are also severely decreased in tumor tissues [17]. Another study showed that hepatocytes isolated from rats treated with the hepatocarcinogen diethylnitrosamine had a reduced rate of MK-4 uptake compared with normal hepatocytes [18]. Des-γ-carboxy prothrombin (DCP), an abnormal prothrombin that is not completely carboxylated, is a well-recognized HCC-specific tumor marker and a predictor of vascular invasion, metastasis and tumor recurrence [1,17,19,20,21]. Notably, DCP production is suppressed by the addition of vitamin K [7,22], and therefore, DCP elevation is thought to result from a deficiency of vitamin K. Recent studies also revealed that DCP functions as a growth and metastasis factor and may contribute to cancer progression [23,24,25,26,27].

**Figure 1:**
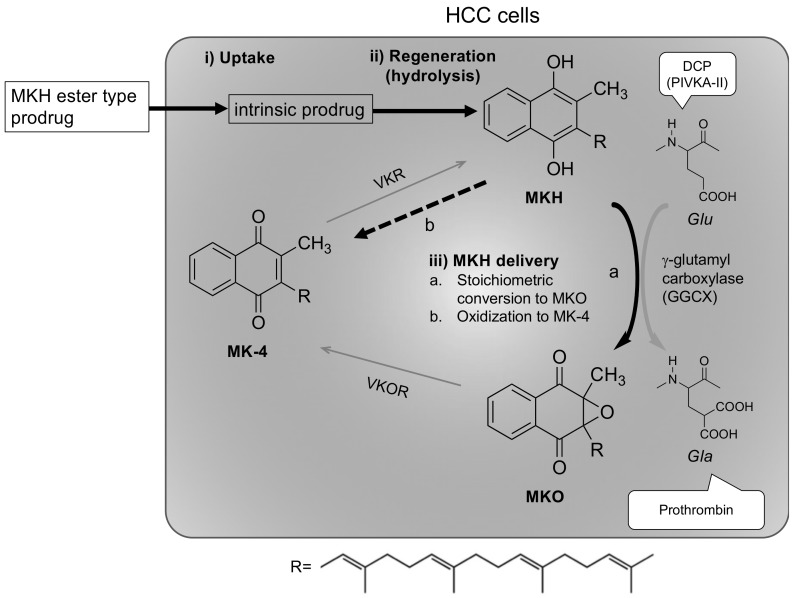
Schematic illustration of the vitamin K cycle and concept of the menahydroquinone-4 (MKH) delivery system. Expected steps of MKH delivery: (**i**) uptake of the MKH ester prodrug; (**ii**) regeneration to parental form MKH; (**iii**) determination of MKH delivery, (a) stoichiometric conversion to menahydroquione-4 epoxide (MKO) and (b) oxidization to menaquinone-4 (MK-4). VKR, vitamin K reductase; VKOR, vitamin K epoxide reductase.

Menahydroquinone-4 (MKH), the reduced form of MK-4, acts as a cofactor of γ-glutamyl carboxylase (GGCX), which converts glutamic acid (Glu) residues to γ-carboxyglutamic acid (Gla) residues in vitamin K-dependent proteins, as shown in Figure 1 [28,29]. In other words, MKH availability regulates the rate of carboxylation. Taken together, these results suggest that decreased MKH availability in HCC cells is one of the possible mechanisms underlying high DCP levels in HCC. We hypothesized that the effective delivery of MKH into HCC cells would be a key to controling HCC growth and metastasis. However, MKH cannot be used as a therapeutic agent owing to its immediate oxidizable characteristics after synthesis. We previously reported that an MKH *N*,*N*-dimethylglycine ester prodrug (MKH-DMG) can deliver the active form effectively into both DCP-positive and -negative HCC cell lines and exhibits a strong growth inhibitory effect compared with MK-4 [22].

**Figure 2:**
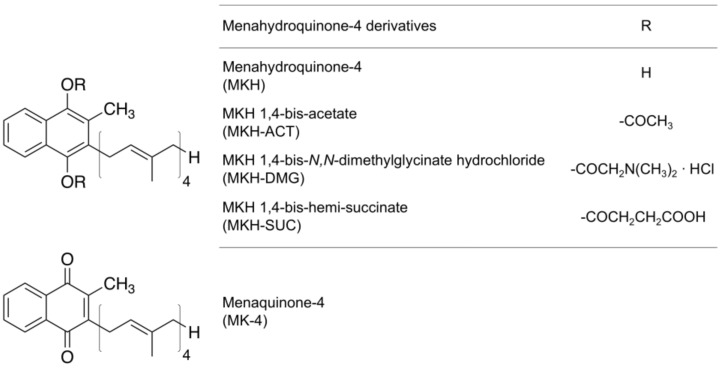
Chemical structures of menahydroquinone-4 (MKH)-ester derivatives and menaquinone-4 (MK-4).

In this study, to expand the possibility of using an MKH prodrug as a chemopreventive agent for the treatment of HCC and to confirm the contribution of each step of the MKH delivery system into HCC in a kinetic study, we prepared anionic MKH succinate (MKH-SUC) and non-ionic MKH acetate (MKH-ACT) as well as cationic MKH-DMG (Figure 2). Succinate and acetate are widely used in commercial preparations of drugs, such as steroids, aspirin and vitamin E [30,31], and their safety and synthesis are thus well-known. We, therefore, chose to investigate the possible uses of these esters in the delivery of MKH. We evaluated the (i) cellular uptake rate; (ii) rate of regeneration compared to the parental form (MKH); (iii) estimation of intracellular MKH; and (iv) antitumor effects in vitro (Figure 1). MKH-SUC showed the highest uptake and the most effective generation of MKH in HCC cell lines among the tested compounds and exhibited rapid and strong antitumor effects in both DCP-positive PLC/PRF/5 and DCP-negative SK-Hep-1 cell lines. These results indicate that the strategy of using an MKH-ester prodrug may be effective for MKH delivery due to the selection of a specific promoiety and controlling of rates of key steps. Our findings demonstrate that MKH-SUC might have a good potential as an MKH delivery system for HCC and overcome the limitations of MK-4 as a clinical chemopreventive agent.

## 2. Results

### 2.1. Uptake of the MKH-Ester Derivatives in HCC Cells

**Figure 3:**
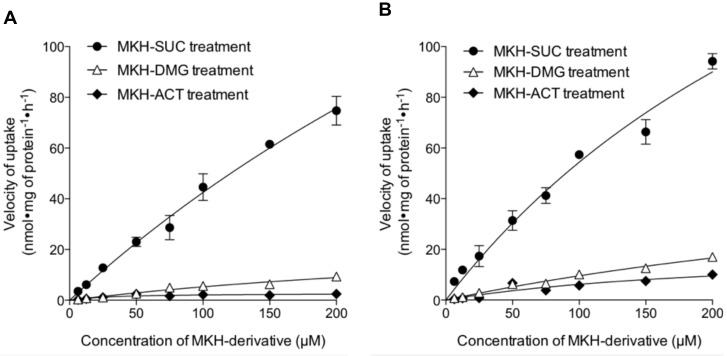
Kinetic plots for the uptake of MKH-ester derivatives in HCC cells. (**A**) PLC/PRF/5 and (**B**) SK-Hep-1 cells were treated with various concentrations of MKH-ACT, MKH-DMG or MKH-SUC. The curves represent the best fit of the Michaelis--Menten equation to the data. Each point represents the mean ± SD from duplicate experiments.

To assess the uptake of the intrinsic MKH-ester derivatives as the first step of MKH delivery into HCC cells, the velocities of uptake of MKH-ester derivatives were plotted and fitted with the Michaelis--Menten model (as shown in Figure 3), and the calculated kinetic parameters were determined (Table 1). All three derivatives increased in both PLC/PRF/5 and SK-Hep-1 cells in a dose-dependent manner. The uptake of MKH-SUC was clearly higher than that of MKH-DMG and MKH-ACT at all concentrations examined. The ratio of the Michaelis--Menten constant (*V*_max_/*K*_m_) for MKH-SUC was about 5- to 10-fold higher than those for MKH-ACT and MKH-DMG in both cell lines.

### 2.2. Regeneration of the MKH-Ester Derivatives to MKH in Homogenized HCC Cells

**Figure 4:**
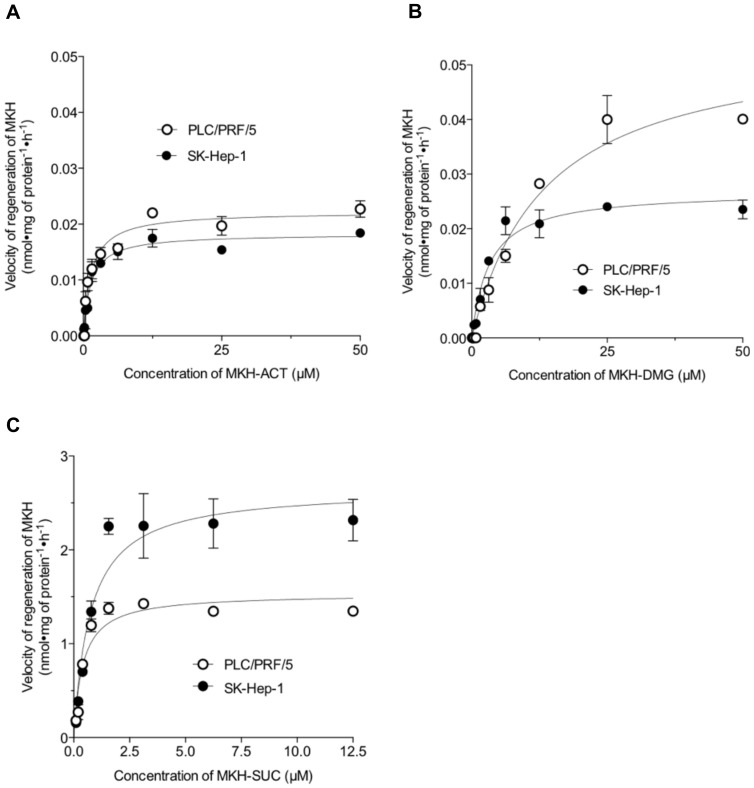
Kinetic plots for the regeneration of MKH-ester derivatives to MKH in S9 fractions of PLC/PRF/5 or SK-Hep-1 cells. (**A**) MKH acetate (MKH-ACT); (**B**) MKH dimethylglycinate (MKH-DMG); and (**C**) MKH succinate (MKH-SUC). Each point represents the mean ± SD from duplicate experiments.

To assess the regeneration to the parental form, MKH, from the MKH-ester derivatives by an esterase of HCC, MKH-ACT, MKH-DMG and MKH-SUC were incubated at 37 °C in S9 fractions obtained from PLC/PRF/5 or SK-Hep-1 cells. The velocities of MK-4 generated in the S9 fractions were plotted as the amount of MKH, because MKH is rapidly oxidized to MK-4 during drug extraction and measurement. The regeneration versus time profile was fitted by the Michaelis--Menten model (shown in Figure 4) and the kinetic parameters were calculated (Table 2). The regeneration velocities of MKH-ACT in both PLC/PRF/5 and SK-Hep-1 cells reached a plateau at about 3 μM MKH-ACT. MKH-DMG reached a plateau in PLC/PRF/5 and SK-Hep-1 cells at 25 μM and about 6 μM MKH-DMG, respectively. MKH-SUC reached a plateau in PLC/PRF/5 and SK-Hep-1 cells at about 0.6 μM and about 1.5 μM MKH-SUC, respectively. The level scale of MKH regeneration from MKH-SUC was about 100-fold higher than those of MKH-ACT and MKH-DMG. The ratio of the Michaelis--Menten constant (*V*_max_/*K*_m_) for MKH-SUC was about 250- to 950-fold higher than those of MKH-ACT and MKH-DMG in both cell lines.

### 2.3. Delivery of MKH into HCC Cells with MKH-Ester Prodrugs

When assessing the delivery of MKH into HCC cells by MKH-ester prodrugs, although the MKH levels in HCC cells should be quantified, it is difficult to determine an accurate MKH level owing to its rapidly oxidative characteristics to MK-4. Concomitant with γ-carboxylation in producing of vitamin K-dependent proteins, MKH is stoichiometrically converted to MK-4 epoxide (MKO), as shown in Figure 1. Thus, MKO levels in HCC cells can reflect the levels of MKH. In addition, the intracellular MK-4 in HCC cells after MKH-DMG treatment is the oxidative product of MKH, which is generated by hydrolysis of MKH-DMG [22]. The estimation rules of MKH in an earlier report were adopted in this study, in which the sum of MKO and MK-4 levels after MKH-ester prodrug treatment was regarded as the MKH level delivered into HCC cells. In MK-4 treatment, the MKH level was assessed by determining the level of MKO.

**Figure 5:**
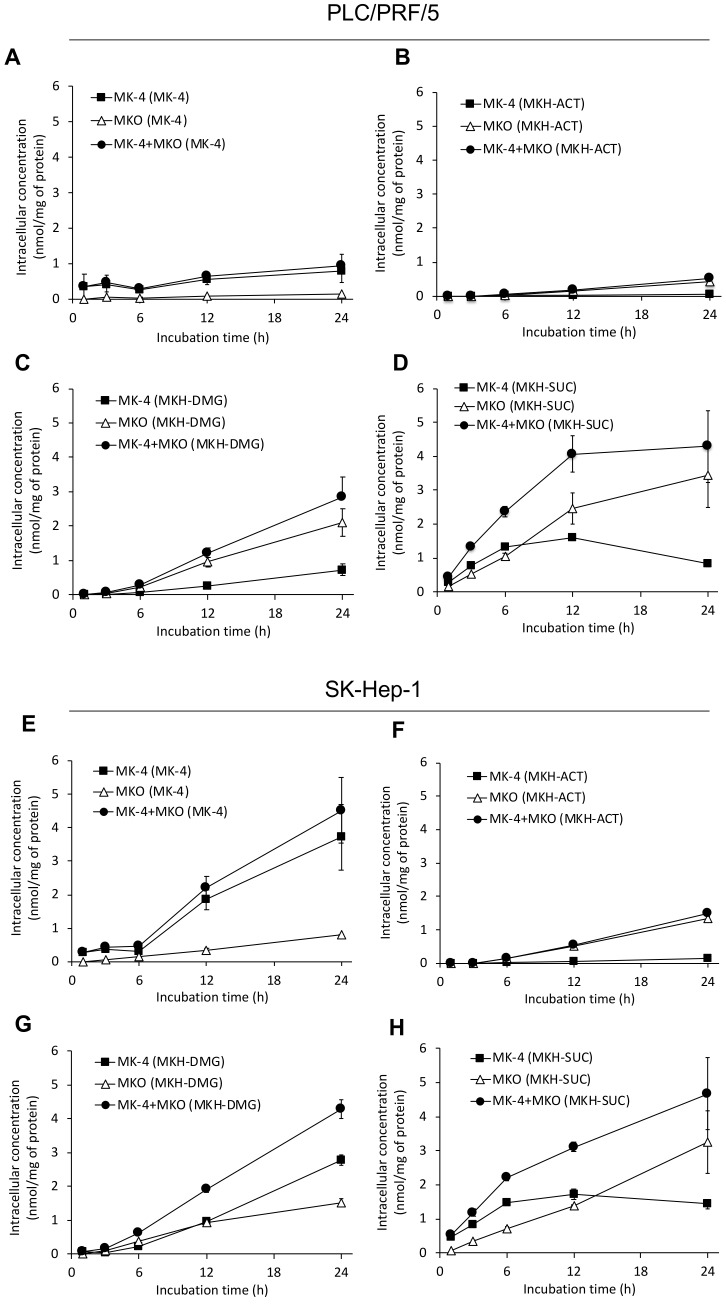
MKH delivery into HCC cell lines via MKH-ester prodrugs or MK-4. Intracellular MK-4 and MKO concentration–time profiles following 25 μM MKH-ester prodrug or MK-4 treatment of PLC/PRF/5 (**A**–**D**) and SK-Hep-1 (**E**–**H**) cell lines. (**A**,**E**), MK-4; (**B**,**F**), MKH-ACT; (**C**,**G**), MKH-DMG; (**D**,**H**), MKH-SUC. Symbols: ■, MK-4; △, MKO; ●, MK-4 + MKO after MKH-ester prodrug or MK-4 treatment. Error bars indicate means ± SDs (*n* = 3).

MKH delivery into HCC cells with 25 μM MKH-ester derivatives was determined after 1, 3, 6, 12 or 24 h of incubation. The dose level was set according to a previous report to ensure that the dose would not strongly affect cell growth and can reflect drug metabolism at around IC_50_ values [22]. After MK-4 treatment, the MK-4 levels gradually increased, but the MKO levels did not increase compared with the MK-4 levels in both cell lines (Figure 5A,E). In contrast, after MKH-ester derivative treatment, the MKO levels efficiently increased in a time-dependent manner compared with each MK-4 level at each time point in both cell lines (Figure 5B–D,F–H). After MKH-SUC treatment, the MK-4 levels in both cell lines reached a plateau after 6 to 12 h of treatment and began to decrease at 24 h (Figure 5D,H). The area under the curve (*AUC*) of the intracellular concentration versus time profile can be used to determine the extent of drug delivery from the administration formulation. The *AUC*_0–24h_ values for MKO (*AUC*_MKO_), MK-4 (*AUC*_MK-4_) and MKH (*AUC*_MKH_) were calculated using the trapezoidal rule (Table 3). In both cell lines, the *AUC*_MKH_ values of each drug were in the order of MKH-SUC > MKH-DMG > MKH-ACT > MK-4. In PLC/PRF/5 cells, the *AUC*_MKH_ values after MKH-ACT, MKH-DMG and MKH-SUC treatments were about 2.3-, 14.2- and 37.1-fold higher than that after MK-4 treatment, respectively. In SK-Hep-1 cells, the *AUC*_MKH_ values after MKH-ACT, MKH-DMG and MKH-SUC treatments were about 1.67-, 5.25- and 7.80-fold higher than that after MK-4 treatment, respectively.

### 2.4. Inhibition of HCC Cell Growth by MKH-Ester Prodrugs

**Figure 6:**
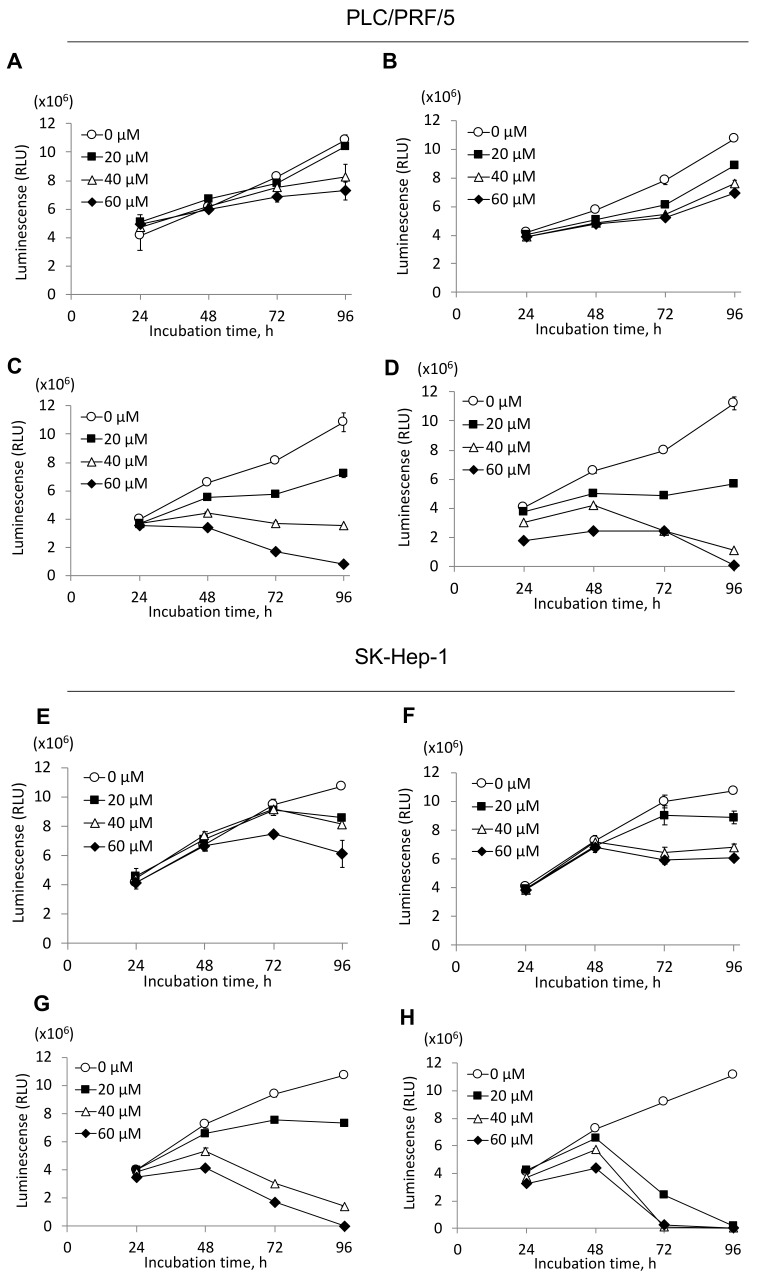
Inhibitory effects of MKH-ester prodrugs and MK-4 on DCP-positive and DCP-negative HCC cell proliferation. MKH-ester prodrugs or MK-4 treatment of PLC/PRF/5 (**A**–**D**) and SK-Hep-1 (**E**–**H**) cell lines. (**A**,**E**), MK-4; (**B**,**F**), MKH-ACT; (**C**,**G**), MKH-DMG; (**D**,**H**), MKH-SUC. PLC/PRF/5 cells are DCP-positive, and SK-Hep-1 cells are DCP-negative. Symbols: ○, 0 μM; ■, 20 μM; △, 40 μM; ◆, 60 μM after MKH-ester prodrug or MK-4 treatment. Error bars indicate means ± SDs (*n* = 3).

To assess the effects of MKH-ester prodrugs on HCC cell proliferation, DCP-positive (PLC/PRF/5) and DCP-negative (SK-Hep-1) HCC cell lines were treated with various concentrations of MKH-ACT, MKH-DMG, MKH-SUC or MK-4 up to 96 h, and cell viability was determined. As shown in Figure 6, in PLC/PRF/5 cells, 40–60 μM MK-4 inhibited about 30–40% of the cell viability of the control at 96 h and maintained the same level of viability of 72 h. In SK-Hep-1 cells, 60 μM MK-4 inhibited about 20% of cell viability of the control at 72 h; further, 20–40 μM MK-4 inhibited 20% of cell viability and 60 μM inhibited 40% of cell viability compared with the control at 96 h. The inhibitory effect of MKH-ACT emerged after 72 h of treatment, and the inhibitory strength was almost the same as that of MK-4. MKH-DMG and MKH-SUC inhibited the proliferation of both HCC cell lines in a time- and dose-dependent manner. MKH-DMG and MKH-SUC showed rapid and strong growth inhibitory effects after 48 h of treatment. The IC_50_ values are summarized in Table 4. In PLC/PRF/5 cells, the IC_50_ values of MKH-ACT, MKH-DMG and MKH-SUC at 96 h of treatment were 0.76-, 3.17- and 4.32-fold lower than that of MK-4, respectively. In SK-Hep-1 cells, the IC_50_ values of MKH-ACT, MKH-DMG and MKH-SUC at 96 h of treatment were 1.23-, 3.61- and 12.3-fold lower than that of MK-4, respectively.

**Figure 7:**
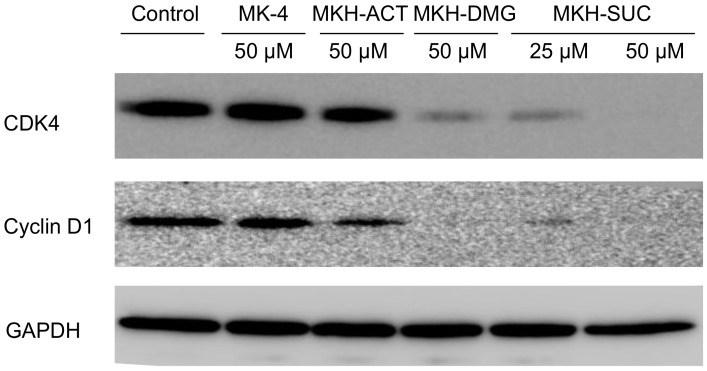
Effects of MKH-ester prodrugs or MK-4 on the expression of the cell cycle regulatory proteins cyclin, D1 and CDK-4, in PLC/PRF/5 cells at 24 h after treatment.

We previously reported that MKH-DMG regulates cell cycle-related proteins as a mechanism of MKH-DMG-induced growth inhibition in PLC/PRF/5, Hep3B and SK-Hep-1 cells [22]. Thus, to clarify the mechanisms of the growth inhibition induced by the MKH-ester prodrugs, we analyzed cell cycle-related proteins using Western blotting (Figure 7). MKH-DMG and MKH-SUC clearly suppressed both cyclin D1 and CDK4 expression compared with controls. MKH-SUC at 50 μM induced a more potent inhibitory effect than 25 μM MKH-SUC on both protein levels. In contrast, MK-4 and MKH-ACT did not affect the expression of the proteins at 24 h after treatment.

## 3. Discussion

We previously demonstrated that the esterified prodrug of MKH, MKH-DMG, can deliver MKH effectively into HCC cells and induce antitumor effects against HCC cells, both in vitro and in vivo [22]. Generally, some chemical modified prodrugs, such as capecitabine and gemcitabine, are activated by specific enzymes in a targeted site, and sometimes, intestinal damage can be prevented owing to the use of inactive prodrug forms. This strategy for MKH-ester prodrugs is not intended to be activated by a unique enzyme of HCC; however, the prodrugs can efficiently deliver MKH because of a carboxy esterase, which is independent of the reductive activation process of MK-4 [22,32]. Li et al. reported that hepatocytes from diethylnitrosamine-induced liver nodules exhibit a significantly lower rate of MK-4 uptake compared with normal hepatocytes [18]. Moreover, chemical modification of the promoiety can produce a substrate of a specific transporter of HCC. In this study, in addition to cationic MKH-DMG, we prepared anionic MKH hemi-succinate (MKH-SUC) and non-ionic MKH acetate (MKH-ACT) and evaluated their MKH delivery in view of the key rate-limiting steps: (i) uptake of the MKH-ester prodrug and (ii) regeneration (hydrolysis) to the parental form, MKH, by an esterase of HCC. The subsequent steps, (iii) MKH delivery and (iv) antitumor effects in HCC cells, were investigated to confirm whether these MKH prodrugs exhibit antitumor effects in a parallel manner to their MKH delivery.

First, the velocity of uptake of the three MKH derivatives into HCC cells was evaluated. MKH-SUC rapidly and highly accumulated into both DCP-positive (PLC/PRF/5) and DCP-negative (SK-Hep-1) cells compared with MKH-ACT and MKH-DMG. The ratio of the Michaelis--Menten constant (*V*_max_/*K*_m_) of MKH-SUC also was about 5- to 10-fold higher than those of MKH-ACT and MKH-DMG. These results clearly show that MKH-SUC has an advantage regarding transport into HCC owing to its anionic promoiety. Although the precise reason underlying the effective uptake needs further study, we speculate that MKH-SUC might be a substrate to some transporters, such as organic anion transporters [33,34].

Second, the velocity of regeneration to the parental form, MKH, of the three MKH-ester derivatives by an esterase of HCC was determined. All three derivatives reached a plateau in a dose-dependent manner. The regeneration velocity of MKH-SUC was the fastest and highest among the three derivatives. The ratio of the Michaelis--Menten constant (*V*_max_/*K*_m_) of MKH-SUC also was about 250- to 950-fold higher than those of MKH-ACT and MKH-DMG. As discussed below, the MKH delivery of MKH-SUC is 4.7- to 15.6-fold higher than that of MKH-ACT, and thus the difference in MKH delivery between MKH-SUC and MKH-ACT may result from the difference in uptake rates.

Next, a time course of MKH delivery with the MKH derivatives, including MK-4, was investigated. The *AUC*_0–24h_ values for MKO after treatment of MKH-SUC was the highest among the three MKH-derivatives in both HCC cell lines, and the *AUC*_0–24h_ values for MKH after treatment of MKH-SUC were also the highest (Table 3). These results reflect the rapid uptake and hydrolysis of MKH-SUC in the HCC cells. However, the difference in MKH delivery was not higher than that of the regeneration to MKH by the esterase of HCC (Figure 4 and Table 2). This suggests that for MKH-SUC, the process of uptake may be the rate limiting step in the delivery of MKH and that the regeneration step was not saturated. For MKH-DMG and MKH-ACT, the rates of uptake and regeneration were not so different; MKH delivery of MKH-DMG was 3.01- to 6.09-fold higher than that of MKH-ACT. These differences may result from the actual amount (nearly equal to *V*_max_ values) of uptake and the regeneration step between MKH-ACT and MKH-DMG, and the rates of transportation/enzymatic reactions to the substances (*K*_m_) of MKH-ACT and MKH-DMG may be sufficient to reach *V*_max_ under the test conditions shown in Figure 5. These results demonstrate that MKH-SUC exhibits good potential as an MKH prodrug owing to its high intracellular uptake and rapid enzymatic activation in HCC cells. Regarding the decrease of MK-4 after 24 h treatment of MKH-SUC in both cells (Figure 5D,H), this phenomenon may be explained by the disposition of hydrolysis of MKH-SUC in an isotonic buffer solution. The half-life of MKH-SUC toward MKH in PBS at 37 °C is about 25 h, and hence, the decrease in MK-4 as an oxidant of MKH reflects the elimination of MKH-SUC in the culture medium. In contrast, MKO almost linearly increased to 24 h despite the elimination of MKH-SUC. This is because, at 24 h, the delivery of MKH, which acts as a cofactor of GGCX, remained sufficient, but excess MKH that would be oxidized to MK-4 may be reduced with the elimination of MKH-SUC.

Finally, to confirm whether these MKH prodrugs have antitumor activity in parallel with their MKH delivery activities, the effects of the MKH-ester prodrugs on HCC cell proliferation were determined. All of the MKH prodrugs and MK-4 inhibited the proliferation of the tested HCC cell lines in a time- and dose-dependent manner. MKH-DMG and MKH-SUC exhibited a strong growth inhibitory effect in both PLC/PRF/5 and SK-Hep-1 cells, while MKH-ACT had little inhibitory effect on cell proliferation. A previous report showed that G1 cell cycle arrest is involved in the anti-proliferative action of MK-4 and MKH-DMG [5,8,22]. In this study, MKH-SUC also suppressed the expression of cyclin D1 and CDK4 at 24 h of treatment, and the effects on protein suppression were consistent with the anti-proliferation results. This result demonstrates that MKH-SUC induces G1 arrest, similar to MKH-DMG and MK-4.

A recent small-scale clinical study reported that the combined use of sorafenib and MK-4 prolonged the survival rate of HCC patients compared with sorafenib alone [27]. Although it was a small clinical trial, there was a clear difference between the two groups of combined and single treatment. Our results show that the MKH prodrug can deliver MKH more efficiently to HCC than MK-4, and thus, a synergistic effect using MKH prodrugs would also be anticipated and may be amplified. Regarding the advantages of these MKH prodrugs, we previously demonstrated that the hydrolysis of MKH-DMG and γ-tocotrienol dimethylglycinate and hemi-succinate is not accelerated by human plasma [35,36]. Moreover, MKH-DMG and MKH-SUC are hydrophilic and can therefore be dispersed in aqueous medium, creating transparent solutions with ethanol and some emulsifiers. These aqueous formulations can then be administered p.o. or i.v. in in vivo experiments [32,35,36].

In conclusion, MKH-SUC can effectively deliver MKH to HCC cells owing to its rapid uptake and reconversion to the parental form, MKH, compared with MKH-ACT and MKH-DMG, and shows strong antitumor activity in HCC cells. Therefore, MKH-SUC may have good potential as an MKH prodrug for HCC. Further, regarding MKH delivery and antitumor activity to PLC/PRF/5 and SK-Hep-1 cells, the results from the three MKH-derivatives supported our hypothesis that effective delivery of MKH is a crucial factor for controlling HCC growth and metastasis. Moreover, this strategy of MKH delivery using MKH-ester prodrugs is a beneficial procedure for regulating the delivery of MKH to HCC cells. After delivery, MKH will be metabolized to its safe form, MK-4. Further studies to clarify the detailed mechanisms of availability of MKH in HCC cells and to translate the effect of MKH prodrugs observed in vitro to a clinical scale, such as an ADME study and pharmacological evaluation in vivo, are required.

## 4. Materials and Methods

### 4.1. General

Measurements of melting points were performed with a micromelting point instrument (Yanagimono, Tokyo, Japan) and are uncorrected. Microanalysis, ^1^H-NMR and mass spectral measurements were performed at the Central Microanalytical Department of Pharmaceutical Sciences, Fukuoka University. The ^1^H-NMR spectra were determined at 500 MHz using a JEOL GX-400 spectrometer (JEOL, Ltd., Tokyo, Japan) in a solution of CD_3_OD with tetramethylsilane as an internal standard. The following abbreviations are used: s = singlet, d = doublet and m = multiplet. Field desorption mass (FD-MS) spectra were determined using a JEOL D-300 spectrometer (JEOL).

### 4.2. Chemicals

MK-4 and menaquinone-4 epoxide (MKO) were kindly provided by Eisai Co., Ltd. (Tokyo, Japan). Menahydroquinone-4 1,4-bis-*N*,*N*-dimethylglycinate hydrochloride (MKH-DMG) was synthesized in our laboratory using a previously reported method [37]. The solvents used for extraction and chromatography were HPLC grade (Wako Pure Chemical Industries, Osaka, Japan).

### 4.3. Synthesis of MKH-Esters

The esters of MKH were obtained by reductive esterification with pyridine as catalyst using a modified, previously described method [32,37], as described briefly below.

#### 4.3.1. Menahydroquinone-4 1,4-Bis Acetate

Menahydroquinone-4 1,4-bis acetate (MKH-ACT): To a dry pyridine solution of MKH (2.25 mmol), reduced by borohydrate from MK-4, 15.75 mmol of acetic anhydride was added. The reaction mixture was stirred at room temperature for 24 h. After the solvent evaporated, the residue was treated with 100 mL of water and then extracted with ethyl acetate (100 mL × 3). The organic layer was dried over anhydrous sodium sulfate and evaporated. The residue was fractionated with a flash column-packed Wakogel LP40, 60A using n-hexane-ethyl acetate (9:1, *v*/*v*) as the eluent. The isolated ester was directly collected in ethyl acetate, and the precipitate was collected and recrystallized from ethanol to give the colorless solid.

Colorless crystal, yield 30%; m.p. 39–41 °C. ^1^H-NMR (MeOH-*d*_4_) δ: menahydroquinone moiety; 7.73 (2H, m, H-5, 8), 7.49 (2H, m, H-6-7), 5.05 (4H, m, H-2’, 6’, 10’, 14’), 3.44 (2H, m, H-1’), 2.24 (3H, s, CH_3_-2), Acetyl moiety; 2.45 (3H, s, CH_3_ 1-ester), 2.42 (3H, s, CH_3_ 4-ester). FD-MS (*m*/*z*); 531 (M^+^ − H). Anal. Calcd. for C_35_H_46_O_4_: C, 79.21; H, 8.74. Found: C, 79.34; H, 8.96.

#### 4.3.2. Menahydroquinone-4 1,4-Bis Hemisuccinate

Menahydroquinone-4 1,4-bis hemisuccinate (MKH-SUC): 18.0 mmol succinic anhydride was added to a dry isopropyl ether–dioxane solution (6:4, *v*/*v*) containing dimethyl amino pyridine of MKH (4.5 mmol), reduced by borohydrate from MK-4. The reaction mixture was stirred at 70 °C for 2 h. After the solvent evaporated, the residue was treated with 100 mL of water and then extracted with ethyl acetate (100 mL × 3). The organic layer was dried over anhydrous sodium sulfate and evaporated. The precipitate was recrystallized from ethyl acetate/n-hexane to give the white solid.

White crystal, yield 16%; m.p. 179–181 °C. ^1^H-NMR (MeOH-*d*_4_) δ: menahydroquinone moiety; 7.89 (2H, m, H-5, 8), 7.47 (2H, m, H-6-7), 5.10 (4H, m, H-2’, 6’, 10’, 14’), 3.48 (2H, d, H-1’), 2.27 (3H, s, CH_3_-2), Succinyl moiety; 2.83–3.13 (8H, m, CH_2_CH_2_ 1, 4-ester). FD-MS (*m*/*z*); 647 (M^+^ − H). Anal. Calcd. for C_35_H_46_O_4_: C, 72.42; H, 7.79. Found: C, 72.28; H, 7.81.

Supplementary materials contain scheme of synthesis of MKH-DMG, MKH-ACT and MKH-SUC and 1H-NMR spectra of MKH-ACT and MKH-SUC.

### 4.4. Cell Lines

The DCP-positive HCC cell line, PLC/PRF/5, was obtained from JCRB Cell Bank (Osaka, Japan). The DCP-negative HCC cell line, SK-Hep-1, was obtained from ECACC (DS Pharma Biomedical). The cell lines were maintained in DMEM (Sigma-Aldrich, St. Louis, MO, USA), supplemented with 10% FBS (Life Technologies, Carlsbad, CA, USA) and 1% penicillin/streptomycin (Life Technologies) at 37 °C under 5% CO_2_ and 95% air.

### 4.5. Cell Viability Assays

Cell viability was determined using a previously reported method [22]. Briefly, PLC/PRF/5 and SK-Hep-1 cells were plated at 5.0 × 10^3^ cells/well in 96-well plates and allowed to attach for 48 h. MK-4 and MKH-ACT were dissolved in 99.5% ethanol. MKH-SUC was dissolved in DMSO. MKH-DMG was dissolved in sterilized water. Stock solutions of each drug (50 mM) were diluted to the intended final concentrations with medium. Cells were exposed to MK-4, MKH-ACT, MKH-DMG or MKH-SUC for up to 96 h, and cell viability was evaluated using the Cell Titer-Glo Luminescent Cell Viability Assay (Promega, Madison, WI, USA) according to the manufacturer’s instructions. IC_50_ values were determined by a log [drug] vs. normalized response-variable slope analysis of GraphPad Prism, version 6.0 (GraphPad Software, San Diego, CA, USA).

### 4.6. Kinetic Analysis of Hydrolysis of MKH-Ester Derivatives

The hydrolysis of the esters was performed at 37 °C in S9 fractions of PLC/PRF/5 and SK-Hep-1 cells after ultrasonic homogenization and centrifugation (10,000× *g*, 1 h) in isotonic phosphate buffer (pH 7.4). Stock solutions of each ester were made in the same manner as the cell viability assay, and the solutions were diluted with 0.1% *w*/*v* albumin in PBS. The enzymatic reactions were initiated by adding 5 μL of the diluted of each ester into amber test tubes containing 95 μL of preheated S9 fractions. A BCA protein assay (PIERCE/Thermo Fisher Scientific Inc., Waltham, MA, USA) was used to determine protein concentrations, and the final S9 concentration was adjusted to 1.0 mg of protein/mL in PBS. The solutions were incubated at 37 °C, and at appropriate times, the aliquots from the reaction were combined with an equal volume of methanol and three times volume of n-hexane. The samples were vortexed for 2 min and centrifuged at 1750× *g* for 10 min. The upper layer (n-hexane) was collected and evaporated under nitrogen. The residue was reconstituted with 100 μL of methanol, sonicated for 10 s and subjected to LC-MS/MS analysis as described below.

### 4.7. Determination of Intracellular MKH Derivatives, MK-4 and MKO, after Drug Treatment

HCC cells were plated at 1.5 × 10^5^ cells/well in 6-well plates and allowed to attach for 48 h. Cells were cultured in medium containing MK-4, MKH-ACT, MKH-DMG or MKH-SUC for various intervals. After the drug exposure, media were removed, and cells were washed three times with PBS. Cells were collected in 1 mL of PBS and sonicated. The cell homogenates were combined with an equal volume of methanol and three times volume of n-hexane, vortexed for 2 min and centrifuged at 1750× *g* for 10 min. The organic layer was evaporated under N_2_ gas. The residue was reconstituted with 100 μL of methanol, sonicated for 10 s and subjected to LC-UV or LC-MS/MS analysis, as described below. The protein concentration of the cell homogenate was determined using a BCA protein assay kit.

### 4.8. LC-MS/MS and LC-UV Analysis

#### 4.8.1. LC-MS/MS

The LC-MS/MS analysis was performed with the same instrument under the same conditions as in an earlier report [22], except for the following MS/MS-multiple reaction monitoring (MRM) setting and retention times: MRM: *m*/*z* 532→171, [M + H]^+^ MKH-ACT adduct; *m*/*z* 664→187, [M + NH_4_]^+^ MKH-SUC adduct; retention times: MKH-ACT, 1.7 min; and MKH-SUC, 1.1 min.

#### 4.8.2. LC-UV

The LC system was the same as that described in the 4.8.1 LC-MS/MS section, and the UV detector was a Prominence SPD-20A (Shimadzu Corp., Kyoto, Japan). Separations were performed on the same column mentioned in the 4.8.1 LC-MS/MS section and monitored at a wavelength of 240 nm. The mobile flow was a binary gradient: (A) 0.1% acetic acid in water and (B) 0.1% acetic acid in methanol. The total flow rate was 0.3 mL/min; 0 min at 80% B, 1 min at 80% B, 4.0 min at 100% B, 7.0 min at 100% B and 8.0 min at 80% B. The Column temperature was 40 °C. The retention times were as follows: MKH-DMG, 9.2 min; MKH-ACT, 8.2 min; and MKH-SUC, 6.6 min.

### 4.9. Western Blotting

HCC cells were plated at 1.5 × 10^5^ cells/well in 6-well plates and allowed to attach for 48 h. Cells were treated with MK-4, MKH-ACT, MKH-DMG or MKH-SUC for 24 h. Western blotting was performed as described previously [22].

### 4.10. Data Analysis

For the determination of *K*_m_ and *V*_max_ for the uptake and enzymatic activation of MKH-esters, concentration-dependency data were fit to the Michaelis--Menten equation:

*V* = *V*_max_·[S]/(*K*_m_ + [S]) (1)

where *V* represents the velocity of action between the substrate and transportation/enzymatic reaction, [S] refers to the concentration of the substrate and *K*_m_ is defined as the concentration of substrate at the half-maximal rate of action (*V*_max_). Data were analyzed by GraphPad Prism (GraphPad Software).

## Figures and Tables

**Table 1:** Kinetic parameters for the uptake of MKH-esters in hepatocellular carcinoma (HCC) cells treated with MKH-ACT, MKH-DMG or MKH-SUC.

Compound	*V*_max_^a^ (nmol·mg of Protein^−1^·h^−1^)	*K*_m_^a^ (μmol·L of Medium^−1^)	*V*_max_/*K*_m_^b^ (10^−3^·L·mg^−1^·h^−1^)	*R* ^2 c^
In PLC/PRF/5 cells				
MKH-ACT	2.76 [1.70 to 3.81]	31.9 [0.0 to 72.3]	0.0865	0.8379
MKH-DMG	28.9 [−4.27 to 62.1]	447 [0.0 to 1124]	0.0647	0.9705
MKH-SUC	345 [84.0 to 606]	712 [61.0 to 1362]	0.485	0.9942
In SK-Hep-1 cells				
MKH-ACT	20.3 [−9.47 to 50.0]	227 [0.0 to 754]	0.0894	0.8601
MKH-DMG	62.6 [4.69 to 121]	556 [0.0 to 1204]	0.113	0.9864
MKH-SUC	261 [74.7 to 448]	381 [8.24 to 753]	0.685	0.9826

^a^ The values are obtained from Michaelis--Menten curve fitting (GraphPad Prism). The values in square brackets indicate 95% confidence intervals. ^b^ Calculated using the best fit values. ^c^ Goodness of fit.

**Table 2:** Kinetic parameters for the regeneration of MKH-ester derivatives to MKH in S9 fractions of PLC/PRF/5 and SK-Hep-1 cells.

Compound	*V*_max_^a^ (nmol·mg of Protein^−1^·h^−1^)	*K*_m_^a^ (μmol·L of Medium^−1^)	*V*_max_/*K_m_*^b^ (10^−3^·L·mg^−1^·h^−1^)	*R* ^2 c^
In PLC/PRF/5 cells				
MKH-ACT	0.0222 [0.0193 to 0.0251]	1.42 [0.643 to 2.19]	0.0156	0.9552
MKH-DMG	0.0550 [0.0435 to 0.0665]	13.6 [6.42 to 20.7]	0.00404	0.9785
MKH-SUC	1.53 [1.25 to 1.81]	0.399 [0.0929 to 0.705]	3.83	0.9144
In SK-Hep-1 cells				
MKH-ACT	0.0182 [0.0164 to 0.0201]	1.32 [0.754 to 1.89]	0.0138	0.9701
MKH-DMG	0.0269 [0.0229 to 0.0308]	3.24 [1.51 to 4.97]	0.00830	0.9674
MKH-SUC	2.65 [2.15 to 3.16]	0.740 [0.215 to 1.27]	3.58	0.9416

^a^ Values are obtained from Michaelis--Menten curve fitting (GraphPad Prism). The values in square brackets indicate 95% confidence intervals. ^b^ Calculated using the best fit values. ^c^ Goodness of fit.

**Table 3:** Area under the intracellular concentration versus time curve (*AUC*) after treatment with MK-4 or MKH-ester prodrugs in HCC cell lines. Doses are 25 μM.

HCC Cell Line	Test Drug	*AUC*_0–24h_ for MKO (nmol·h·mg Protein^−1^)	*AUC*_0–24h_ for MK-4 (nmol·h·mg Protein^−1^)	*AUC*_0–24h_ for MKH ^a^ (nmol·h·mg Protein^−1^)
PLC/PRF/5	MK-4	2.07 ± 0.308	12.2 ± 4.19	2.07 ± 0.308
	MKH-ACT	4.05 ± 0.460	0.784 ± 0.107	4.83 ± 0.396
	MKH-DMG	22.4 ± 3.92	6.99 ± 1.15	29.4 ± 5.02
	MKH-SUC	48.9 ± 10.6	27.6 ± 1.66	76.7 ± 11.9
SK-Hep-1	MK-4	8.80 ± 0.467	41.6 ± 9.04	8.80 ± 0.467
	MKH-ACT	13.3 ± 0.721	1.44 ± 0.177	14.7 ± 0.898
	MKH-DMG	19.6 ± 1.44	26.6 ± 1.37	46.2 ± 2.79
	MKH-SUC	36.2 ± 5.92	33.3 ± 2.42	69.5 ± 8.03

^a^ MKH value after MKH-ester prodrug treatment: sum of MKO and MK-4; MKH value after MK-4 treatment: MKO.

**Table 4:** The IC_50_ values of MK-4 and MKH-ester derivatives in PLC/PRF/5 or SK-Hep-1 cells for the different incubation time periods.

Time (h)	IC_50_ Value (μM) ^a^			
	PLC/PRF/5			
	MK-4	MKH-ACT	MKH-DMG	MKH-SUC
48	Not calculated	Not calculated	63.04 [55.67–71.38]	48.86 [30.78–77.56]
72	188.3 [80.86–438.6]	151.9 [35.69–646.4]	33.4 [25.57–43.61]	26.59 [14.74–47.97]
96	86.84 [45.85–164.5]	114.5 [71.49–183.3]	27.38 [20.65–36.31]	20.12 [18.68–21.67]
	SK-Hep-1			
	MK-4	MKH-ACT	MKH-DMG	MKH-SUC
48	Not calculated	Not calculated	71.3 [70.72–71.90]	78.13 [61.53–99.21]
72	80.16 [47.51–135.2]	71.58 [34.92–146.8]	32.45 [27.38–38.46]	15.8 [11.56–21.60]
96	88.19 [25.15–309.2]	71.94 [45.98–112.6]	24.4 [22.39–26.58]	7.161 [3.671–13.97]

^a^ Best fit values of IC_50_ were obtained by Graph Pad Prism, and the 95% confidence intervals are shown in the square brackets.

## Supplementary Materials

The following are available online at http://www.mdpi.com/1420-3049/23/7/1738/s1, Figure S1: Scheme of syntheses of MKH-DMG, MKH-ACT and MKH-SUC, Figure S2: ^1^H-NMR spectra of MKH-ACT and MKH-SUC.
