# Supplementary material for: Antitumor Effects and Delivery Profiles of Menahydroquinone-4 Prodrugs with Ionic or Nonionic Promoiety to Hepatocellular Carcinoma Cells
Source: Molecules. 2018 Jul 16;23(7):1738. doi: 10.3390/molecules23071738 (PMC6100056; doi:10.3390/molecules23071738)
Supplement: Supplementary file 1 [file molecules-23-01738-s001.pdf]

Supplementary Materials

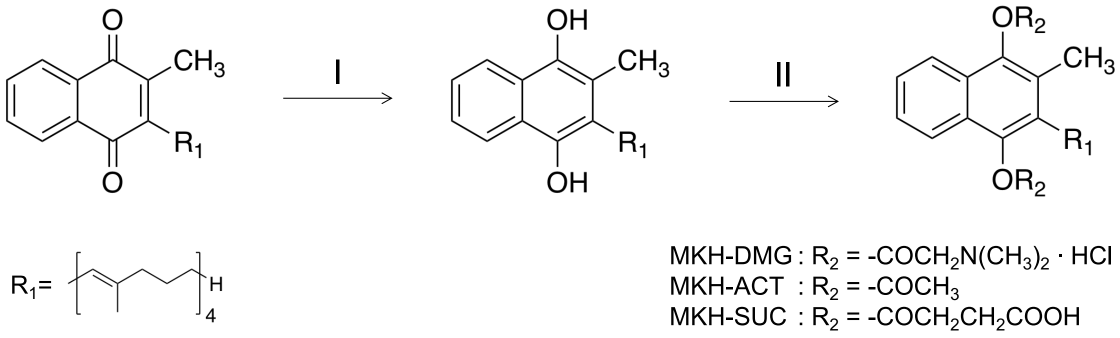

Figure S1. Scheme of syntheses of MKH-DMG, MKH-ACT and MKH-SUC. Reagents and conditions: (I) $NaBH_4$, room temperature (r.t.); (II) *N*,*N*-dimethylglycine hydrochloride, DCC, dry pyridine, r.t. (**MKH-DMG**); acetic anhydride, dry pyridine, r.t. (**MKH-ACT**); succinic anhydride, dimethyl amino pyridine in dry isopropyl ether-dioxane solution (6:4, v/v), 70°C (**MKH-SUC**).

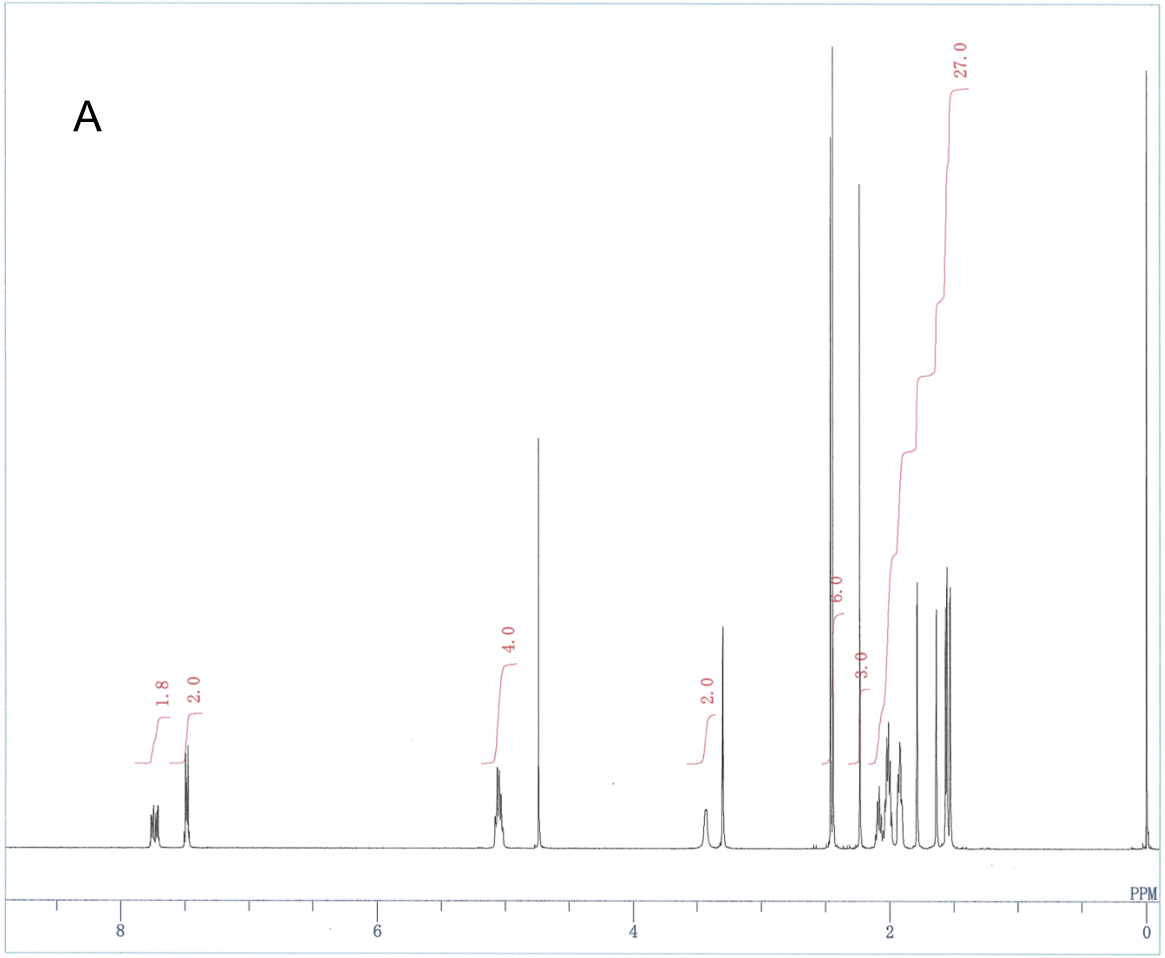
A
1.8
2.0
4.0
2.0
6.0
3.0
27.0
PPM
8
6
4
2
0

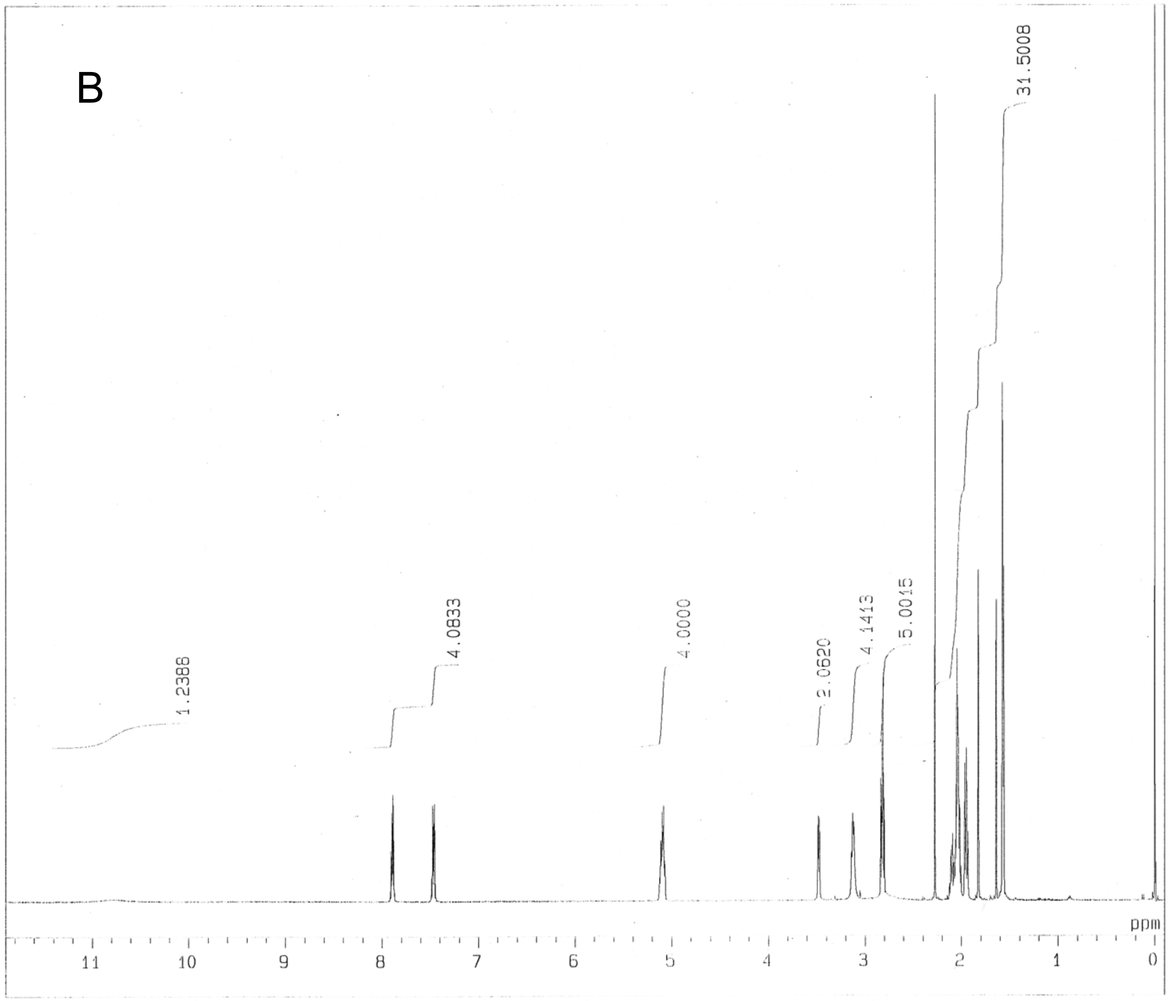

Figure S2. ^{1}H-NMR spectra of MKH-ACT (A) and MKH-SUC (B).
